# Actinomyces odontolyticus Infection of a Marsupialized Vallecular Cyst in a Two-Month-Old Infant: A Report of a Rare Case

**DOI:** 10.7759/cureus.113877

**Published:** 2026-08-03

**Authors:** McKenzie Johnson, Sarah Labuda, Conor Smith, Sara C Sanders, Dustin Williford, Adam Johnson, Jacob Filipek

**Affiliations:** 1 Pediatrics, Le Bonheur Children's Hospital, Memphis, USA; 2 Infectious Diseases, Arkansas Children's Hospital, Little Rock, USA; 3 Pediatrics/Hospital Medicine, University of Arkansas for Medical Sciences, Little Rock, USA; 4 Otolaryngology, University of Arkansas for Medical Sciences, Little Rock, USA; 5 Hospital Medicine, University of Arkansas for Medical Sciences, Little Rock, USA

**Keywords:** actinomyces, actinomyces odontolyticus, otolaryngology case report, pediatric infectious disease, treatment options, vallecular cyst

## Abstract

*Actinomyces* is a rare cause of cervicofacial infections, particularly in children. Children with *Actinomyces* infection often have delayed diagnoses due to low culture sensitivity and variable and indistinct presentations that resemble other pathologies, such as malignancy or tuberculosis. Once the diagnosis is made, a prolonged course of antibiotics is required, often in addition to surgical debridement or drainage. We report a case of a vallecular cyst with superimposed *Actinomyces* infection in a two-month-old girl. The low prevalence of *Actinomyces* infections and the variable non-specific presentations can delay diagnosis and treatment of *Actinomyces* infections.

## Introduction

*Actinomyces* are Gram-positive filamentous anaerobic bacteria that colonize the oral cavity as part of the normal flora [1,2]. Actinomycosis is a rare endogenous infection resulting from access to deeper tissues via trauma, surgical procedures, or foreign bodies that disrupt the mucosal surface [3]. Once invaded, the commensal bacteria then become pathogenic, typically forming masses that spread contiguously across tissue planes. *Actinomyces* can also form sinus tracts that spontaneously heal and recur [4].

The prevalence of actinomycosis is not well studied, but it is considered an uncommon disease, with pediatric cases being much less frequent than in adults [3]. In a retrospective cohort study, infections with *Actinomyces* in children and adolescents had a median age of 19 years, with the majority being adolescents [5]. Cervicofacial infection is the most common manifestation of actinomycosis, though it can infect almost any organ [2,6]. Most cases are indolent, slowly progressive courses that are usually painless at first. In rare cases, the course of infection can be rapid and fulminant [2]. The latter is more highly associated with immunocompromised patients [2]. It is vital to treat with high doses of penicillin for a prolonged period to extend past the resolution of the infection in order to minimize risk of recurrence, which is seen in about one-third of patients within one year of initial presentation [5,7].

Pathognomonic findings include large abscesses that can be connected by sinus tracts that express the typical thick, yellow exudate with characteristic sulfur-like granules [2]. These granules are masses of the filamentous organisms bound by calcium salts and biofilm [2]. Diagnosis can be made by abscess culture for anaerobes [2]. Briefly, vallecular cysts are rare congenital anomalies of the larynx, with an estimated incidence of 1.89-3.49 per 100,000 live births [8]. Vallecular cysts are located between the base of the tongue and the lingual surface of the epiglottis, arising from obstruction of mucous-secreting glands [8]. Our case is exceedingly rare, as our patient had actinomycosis of a vallecular cyst.

## Case presentation

A previously healthy two-month-old girl initially presented to a large southeastern children’s hospital with a temperature of 100.9 degrees Fahrenheit (°F). She was a full-term baby with no pregnancy or birth complications. She was admitted to the hospital for two days for dehydration and sepsis workup, during which she received IV fluids and ceftriaxone at meningitic dosing, had a negative respiratory pathogen panel as well as blood, urine, and CSF cultures, and improved clinically. 

**Figure 1:**
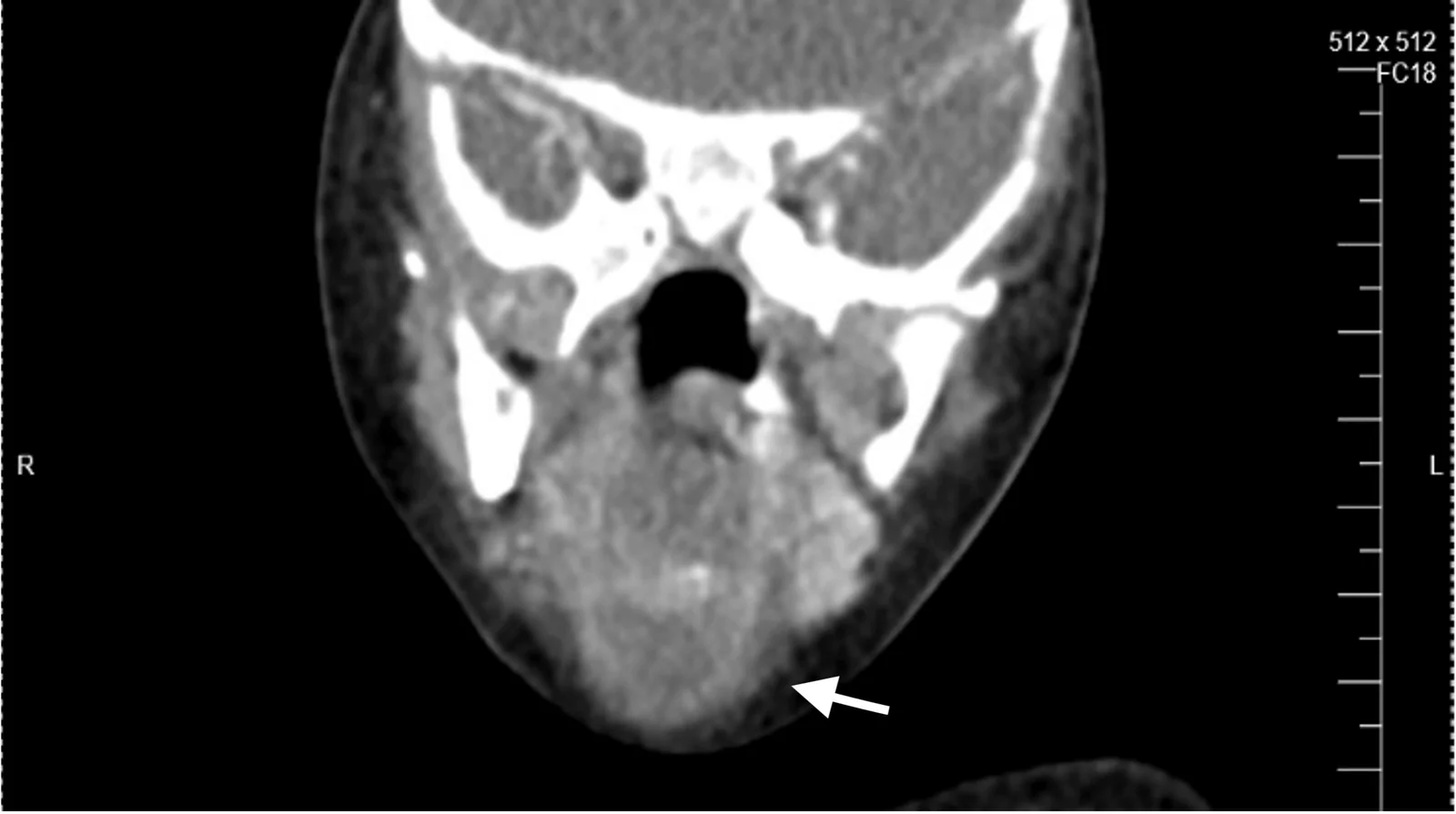
Coronal computed tomography (CT) image of the vallecular cyst (arrow) before drainage.

**Figure 2:**
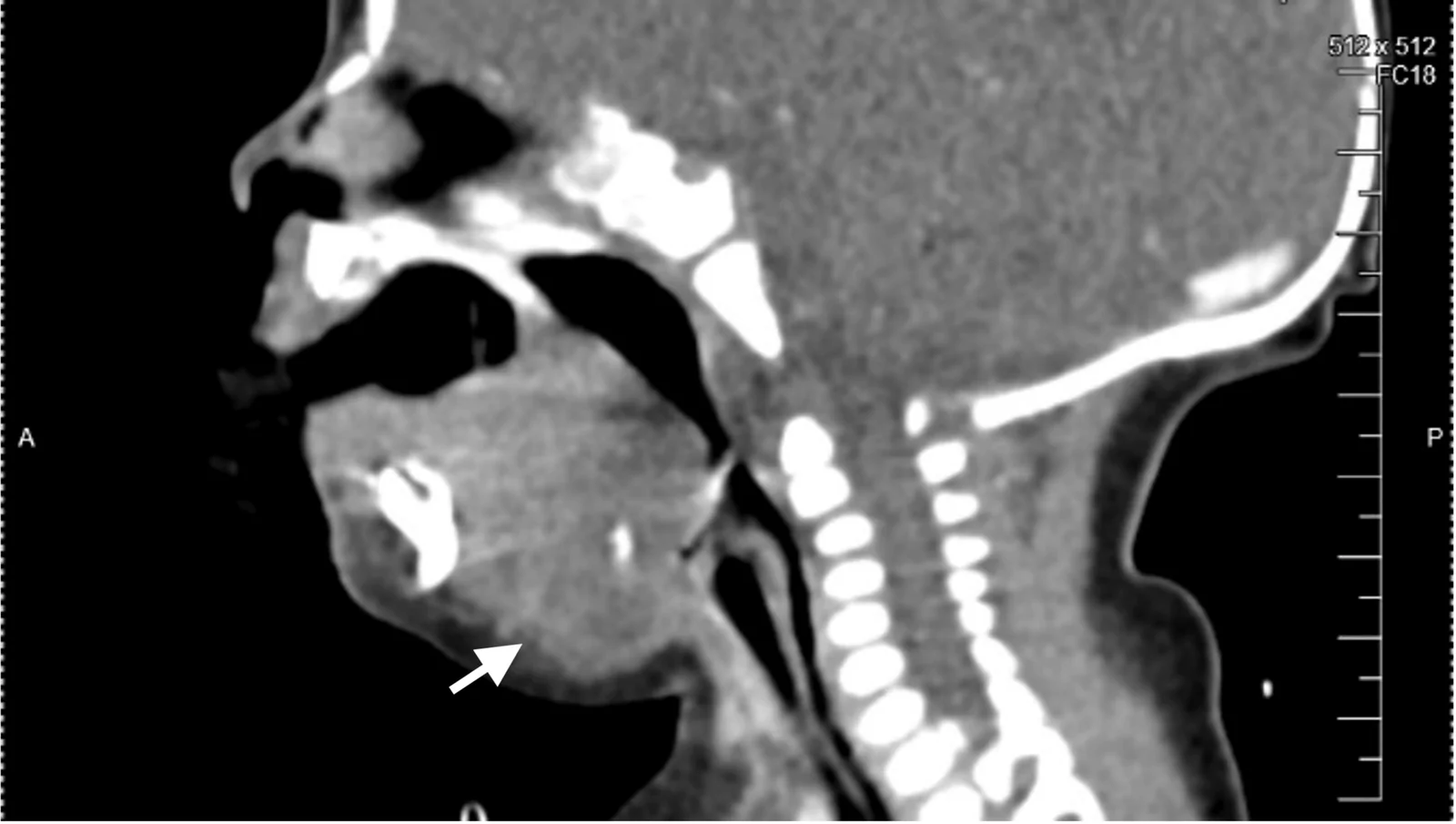
Sagittal computed tomography (CT) image of the vallecular cyst (arrow) before drainage.

One day after discharge, she returned to the hospital with congestion, rhinorrhea, coughing, and choking with feeds (a new finding not noted during previous admission). She was tachypneic and febrile to 101.4°F. On physical exam, there was a protruding midline submental mass (not previously noted during previous admission). A Speech Language Pathologist was consulted in the setting of choking episodes and, on evaluation in the Emergency Department, noted audible stertor and wheezing after feeding with thin liquids; it was also noted that the patient was a pacifier user. A head and neck computed tomography (CT) was performed, and the results were consistent with a vallecular cyst as shown in Figures 1-2. Otolaryngology (ENT) was consulted, and the patient was admitted for observation.

**Figure 3:**
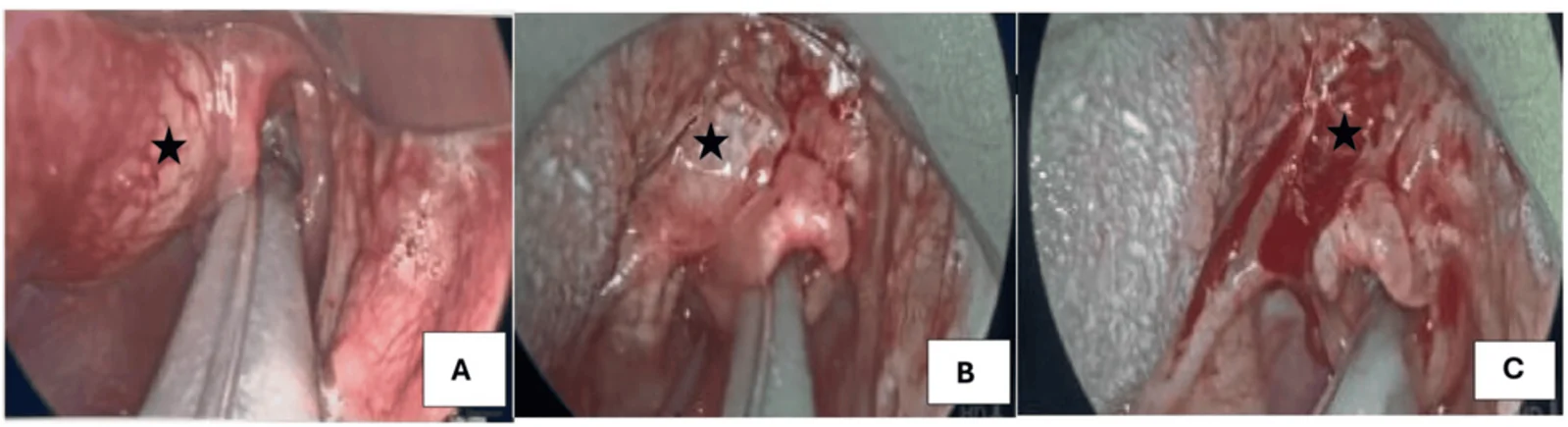
Intraoperative images of the vallecular cyst: (A) Vallecular cyst before drainage (star). (B) Vallecular cyst after drainage (star). (C) Post-marsupialization (star).

Shortly after admission to the general pediatric inpatient floor, the patient began having increased respiratory distress progressing to respiratory failure, requiring emergent intubation by ENT during attempted fiberoptic laryngoscopy, resulting in transfer to the pediatric intensive care unit (PICU). She remained intubated for about 36 hours until ENT extubated her in the operating room (OR). An urgent microlaryngoscopy with incision and drainage (I&D) of a vallecular cyst, classified by its location in the larynx, was performed, and an abscess was found; purulence was expressed and sent for culture. Marsupialization, a surgical technique used on cysts that involves partial removal of the cyst lining, was performed after the I&D to ensure that reformation of the cyst did not occur, as shown in Figure 3. She remained stable on room air and remained extubated throughout the remainder of the admission.

The culture from the I&D of the abscess was positive for *Actinomyces odontolyticus*. She was started on IV ampicillin/sulbactam for four weeks via a peripherally inserted central catheter (PICC) line; at discharge, she was transitioned to oral amoxicillin for six months. She had outpatient follow-up appointments with ENT as well as Infectious Diseases at two weeks and four weeks after discharge, respectively.

At both follow-ups, she presented well with no neck mass, fever, cough, or difficulty eating or breathing. She continued to gain weight appropriately and continued taking the oral amoxicillin. A neck magnetic resonance imaging (MRI) study performed two months after discharge demonstrated near-complete resolution of the previously identified vallecular cyst, with no interval fluid collection.

## Discussion

The age of the patient in this case is particularly interesting since only about 30% of infants at two months of age already have *A. odontolyticus* colonized in their oropharynx [3]. Though the overall prevalence of actinomycosis has not yet been determined, pediatric cases are a very small portion of the rare actinomycosis cases reported. Only 2.6% of all confirmed cases affected patients younger than 10 years old in one study [6]. In addition to young age for this infection, no known cause of mucosal injury (trauma, erupting teeth, surgical procedures, etc.) was identified, which makes the pathogenesis of this case even more curious.

Cervicofacial is the most common site of *Actinomyces* infection, constituting about 60% of all cases. However, as of 2020, there were only 28 cases of pediatric cervicofacial actinomycosis reported in the literature [9]. Most commonly, it presents as a progressive, painless multicystic mass with or without discoloration of overlying skin. The location of vallecular cysts residing at the base of the tongue at the laryngeal inlet can cause airway obstruction as well as feeding difficulties. Difficulty eating and breathing may occur in late stages of infection [10-12]. These infections can rapidly progress, causing fatal conditions such as airway obstruction, as seen in this case, likely due to an immature immune system that could not minimize the spread of infection via granuloma formation [10]. Evaluation requires clinical suspicion since there are no specific serologic or image findings. Though culture is the gold standard of diagnosis, it can be falsely negative up to 50% of the time [10]. Gram-staining is a valuable tool in initial work-up as it will show the characteristic branched, beaded, Gram-positive filamentous rods [10]. The prognosis after prolonged antibiotic treatment is excellent [10]. First-line therapy includes high-dose penicillin G with an extended (6-12 month) course of oral amoxicillin or cephalosporins [2]. Surgical resection may also be indicated depending on the severity, such as in our case. Response to treatment can be monitored with subsequent imaging [10].

Without proper diagnosis and treatment, serious and life-threatening complications can arise. Osteomyelitis is highly associated with pediatric cervicofacial *Actinomyces* (41%) [1]. Other complications include abscesses, endocarditis, hepatic actinomycosis, and CNS involvement, including meningitis, brain abscess, actinomycetoma (nodules, scars, abscesses, and fistulae that drain serous or purulent material containing *Actinomyces* [13]), spinal epidural infection, and cranial, epidural, and subdural infections [10]. These CNS involvements are associated with high mortality rates [4]. Actinomycosis is a rare disease process that often requires intervention from multiple specialists, further complicated by the findings of a vallecular cyst, as illustrated in our case.

## Conclusions

Actinomycosis is a rare and difficult-to-diagnose disease based on its insidious nature and non-specific symptoms. Early treatment with a prolonged course of antibiotics has an excellent prognosis, whereas delayed diagnosis can lead to serious complications that increase morbidity and mortality. As such, this case demonstrates a rare, easy-to-miss diagnosis with significant consequences when not considered early in diagnostic workup. It was further complicated by the presence of a vallecular cyst, which can mimic more common pediatric conditions, such as stridor associated with laryngomalacia.

Because of the benefit of early treatment and risk of complications from untreated actinomycosis, Infectious Diseases consultation for appropriate management along with surgical or surgical subspecialty consult for surgical source control is encouraged to improve infection management and hopefully reduce morbidity/mortality risk in this patient population.
